# Phenotypic Profiling of *Scedosporium aurantiacum*, an Opportunistic Pathogen Colonizing Human Lungs

**DOI:** 10.1371/journal.pone.0122354

**Published:** 2015-03-26

**Authors:** Jashanpreet Kaur, Shu Yao Duan, Lea A. I. Vaas, Anahit Penesyan, Wieland Meyer, Ian T. Paulsen, Helena Nevalainen

**Affiliations:** 1 Department of Chemistry and Biomolecular Sciences, Macquarie University, Sydney, Australia; 2 Biomolecular Frontiers Research Centre, Macquarie University, Sydney, Australia; 3 Molecular Mycology Research Laboratory, Centre for Infectious Diseases and Microbiology, Marie Bashir Institute for Infectious Diseases and Biosecurity, Sydney Medical School—Westmead Hospital, The University of Sydney, Westmead Millennium Institute, Sydney, Australia; 4 Bioinformatics Group, Centralbureau voor Schimmelculturen—Fungal Biodiversity Centre, Utrecht, The Netherlands; Beijing Institute of Microbiology and Epidemiology, CHINA

## Abstract

Genotyping studies of Australian *Scedosporium* isolates have revealed the strong prevalence of a recently described species: *Scedosporium aurantiacum*. In addition to occurring in the environment, this fungus is also known to colonise the respiratory tracts of cystic fibrosis (CF) patients. A high throughput Phenotype Microarray (PM) analysis using 94 assorted substrates (sugars, amino acids, hexose-acids and carboxylic acids) was carried out for four isolates exhibiting different levels of virulence, determined using a *Galleria mellonella* infection model. A significant difference was observed in the substrate utilisation patterns of strains displaying differential virulence. For example, certain sugars such as sucrose (saccharose) were utilised only by low virulence strains whereas some sugar derivatives such as D-turanose promoted respiration only in the more virulent strains. Strains with a higher level of virulence also displayed flexibility and metabolic adaptability at two different temperature conditions tested (28 and 37°C). Phenotype microarray data were integrated with the whole-genome sequence data of *S*. *aurantiacum* to reconstruct a pathway map for the metabolism of selected substrates to further elucidate differences between the strains.

## Introduction

*S*. *aurantiacum* is a ubiquitous ascomycetous fungus found in diverse ecological niches including soil, sewage and polluted waters [1]. It has been recently added to the *S*. *boydii* species complex as a subset of isolates previously identified as *Scedosporium apiospermum* [2–5]. This emerging pathogen has been reported to be less susceptible to antifungals than other members of the *S*. *boydii* complex, such as *S*. *apiospermum* [6–8]. *S*. *aurantiacum* is an opportunistic pathogen capable of causing a wide variety of localized and superficial infections, such as malignant otitis externa, osteomyelitis, invasive sinusitis, keratitis and pneumonia [9, 10]. While *S*. *aurantiacum* has been associated with airway colonization in Europe, *S*. *aurantiacum* related infections have been reported mainly in Australia [9, 11]. Recent population-based surveys have indicated that 17.4% of sputum specimens of Australian cystic fibrosis (CF) patients contain *S*. *aurantiacum*. This makes *S*. *aurantiacum* the second most common filamentous fungus associated with CF in Australia after *Aspergillus fumigatus* [12, 13]. CF, a genetically inherited disease, is characterized by defective mucociliary clearance, which provides an ideal environment for the growth of airborne fungal conidia in the lung [14]. The colonization of the respiratory tracts of Australian CF patients by *S*. *aurantiacum* can possibly be attributed to its relative high abundance in the Australian environment [2].

Considering the increasing incidences of *Scedosporium* infections, and high mortality rate associated with CF, there is a need to develop treatment strategies for these fungal infections [11]. The successful development of preventative strategies is limited by the similarity between the mammalian and fungal cell structures and metabolic pathways. The majority of the work reported on *S*. *aurantiacum* features clinical case studies and epidemiology research, with no published literature on the physiology and biochemistry of the organism [15]. Therefore, studies relating to cell growth, viability and general metabolism can provide a good starting point to facilitate the identification of novel targets to inhibit fungal growth without affecting the human host [15, 16].

As individual cell-based growth assays are relatively slow and can be used to test only a few phenotypes at a time [17], high throughput systems have been devised for the profiling of nutrient utilization in microorganisms [16]. One such method is a phenotype microarray (PM) carried out in 96-well microtitre plates, containing a variety of nutrients (*e*.*g*. sugars), where cell viability and respiration is automatically recorded [18]. Carbon utilization profiles in some filamentous fungi including *Aspergillus*, *Neurospora*, *Hypocrea* and *Acremonium* have been studied with this approach [16, 19–21].

In this study, we have evaluated the phenotypes of four *S*. *aurantiacum* strains isolated from clinical and environmental sources by recording their respiration rates on 94 substrates in microtitre plate assays. The results generated from respiration-based PM assays were validated by shake flask cultivation of the strains on selected carbon sources. Data obtained from the PM assays were compared against the *S*. *aurantiacum* draft genome for the presence or absence of particular metabolism related genes. The *Galleria mellonella* larvae models were used to assess the virulence levels of the four *S*. *aurantiacum* strains studied in this work.

## Materials and Methods

### *Scedosporium aurantiacum* strains

*Scedosporium* strains selected for the studies were obtained from the culture collection of the Medical Mycology Research Laboratory, Centre for Infectious Diseases and Microbiology, Westmead Hospital, Sydney, Australia and included: (1) WM 06.482 isolated from the broncho-alveolar lavage of a cystic fibrosis patient in Australia; (2) WM 09.24 isolated from Sydney Circular Quay [7, 22]; (3) WM 08.202 (FMR8630; CBS116910) a type strain of *S*. *aurantiacum* isolated from a wound exudate of a patient in Santiago de Compostela, Spain and originally sourced from CBS culture collection (CBS-KNAW Fungal Biodiversity Centre, Netherlands), and (4) WM 10.136 (INS1120) isolated from a valley near Innsbruck, Austria. All the strains are a part of the Australian and global MLST (Multilocus Sequence Typing) network. Potential virulence of two of the *S*. *aurantiacum* strains addressed in this work (WM 06.482 and WM 08.202) has previously been assessed in an immunocompetent mouse model by Harun *et al* [7].

### Growth measurements

All *S*. *aurantiacum* strains were cultured on Sabouraud dextrose agar plates (BD, Difco^TM^, Australia) for 5 days at 37°C to achieve sufficient growth and conidiation. Three independent plate cultures were maintained for each strain in order to establish the differences in the appearance of colonies. Conidia were harvested from the cultures using 5 ml of sterile saline (0.9% w/v NaCl and 0.01% v/v Tween 80). The suspension was then filtered through sterile glass wool to separate conidia from the hyphae. The concentration was adjusted to 1x10^6^ conidia/ml for inoculation of liquid cultures each containing 50 ml of Sabouraud’s broth (Sigma Aldrich, Australia) in a 250 ml conical flask. Different flasks were maintained for each time point and all the cultures were incubated at both 28 and 37°C on an orbital shaker at 250 rpm for a total of seven days. The mycelia were collected from the growing cultures by taking out one flask after every 12 hours, filtering the content through pre-weighed Whatman filter paper number 1 and drying in a vacuum oven at 70°C to a constant weight. Dry weight was calculated as the difference between the weight of the filter paper with and without the mycelia.

### Pathogenicity testing using a *Galleria mellonella* larval model

Pathogenicity of the four *S*. *aurantiacum* strains was assessed using the invertebrate *G*. *mellonella* infection model [23]. Conidia were washed twice and diluted in PBS (phosphate-buffered saline) to a final concentration of 10^6^ conidia/ml in an inoculum. *G*. *mellonella* larvae were obtained after the oviposition of the adult moths reared and maintained at 26°C and 60% relative humidity in the insectarium of the Westmead Hospital Animal Care Facility, Sydney, Australia. Ten similar sized larvae were weighed (about 3000 mg each) and placed in a 90 mm plastic Petri dish. Fungal inoculum (10 μl) was then injected into the last left pro-leg of the hemocoel of each larva using a 50 U syringe with a 29-gauge needle. Two different controls were also included in the assay: a group of 10 larvae inoculated with PBS to monitor potential effects on survival due to physical injury, and 10 untreated larvae. After injection, the larvae were incubated in Petri dishes at 35°C for 10 days and checked daily for survival. Larvae were considered dead when they were dark coloured and failed to respond to physical stimuli applied with a forceps. Survival of the larvae against each fungal strain was plotted after performing statistical analysis using Graph Pad Prism 6 (La Jolla, CA, USA).

### Phenotype microarray

Biolog Phenotype analysis was carried out for all four *S*. *aurantiacum* strains using GEN III MicroPlate^TM^ (Biolog Inc, USA) containing 94 assorted substrates (sugars, amino acids, hexose acids and carboxylic acids) and a positive and negative control (S1 Fig.). Fungal conidial suspensions (1x 10^5^/ml) were prepared in the inoculating fluid (IF, Biolog, USA) and 100 μl of the inoculum was dispensed in three replicates into each well of the plate using a multichannel pipette (Biolog). After inoculation, the plates were incubated in the OmniLog incubator/reader (Biolog) for 72 hours at 28°C and 37°C. Cell respiration was recorded every 15 minutes by a charge-coupled device camera and plotted as a kinetic curve depicting reduction of the colorless tetrazolium blue dye to violet (formazan) by cell respiration. Raw values were imported from the OmniLog reader and analyzed using R package software opm [24]. This resulted in two datasets which comprised four strains x three replicates x two measurement temperatures x 94 substrates giving rise to 2256 individual cell respiration curves. Classification of phenotypes was performed based on the maximal curve height calculated as Omni Log units; an Omni Log value greater than 100 was considered as a positive phenotype. Comparison of substrate utilization in different strains was carried out using heat maps, which classified the strains based on estimation of the maximum height of the cell respiration curve [24].

### Correlation of growth and respiration rates

Growth as biomass formation was determined for the four *S*. *aurantiacum* strains in shake flask cultivations on selected carbon sources also used in the Biolog respiration assay. Fungal conidia (1 x 10^5^/ml) were inoculated into 50 ml of M9 minimal medium (Sigma), supplemented with a selected carbon source (1 mM) in a 250 ml shake flask and incubated at 37°C and 200 rpm for 60 hours. Carbon sources tested included maltose, D-trehalose, sucrose, D-turanose, D-salicin, D-glucose and D- fructose. All compounds were obtained from Sigma-Aldrich, dissolved in sterile water and filter sterilized. Cell dry weight was measured at the end of the incubation period as described above, and plotted against the OmniLog units obtained from the respiration curves. Each compound was tested in biological triplicate.

### Metabolic pathway analysis and genome correlation

Sequencing of the genomes of the four *S*. *aurantiacum* strains used in this study has been completed recently with pending annotation [25]. Therefore, information on the putative pathways and enzymatic steps involved in the metabolism of the selected sugars was extracted from KEGG and MetaCyc and additional literature searches [26]. Amino acid translations of the gene sequences involved in the metabolic pathways of interest were identified from closely related reference organisms including *Trichoderma reesei*, *Aspergillus fumigatus*, *Penicillium chrysogenum and Neurospora crassa* and mapped back into the *S*. *aurantiacum* genome data using the tblastn program of BLAST algorithm [27]. Biological function was assigned for each gene encoding a particular enzyme in the predicted pathway based on the homology between the genome sequences for each *S*. *aurantiacum* strain and reference gene sequences [28, 29].

## Results and Discussion

### Growth pattern of *Scedosporium aurantiacum*

**Fig 1:**
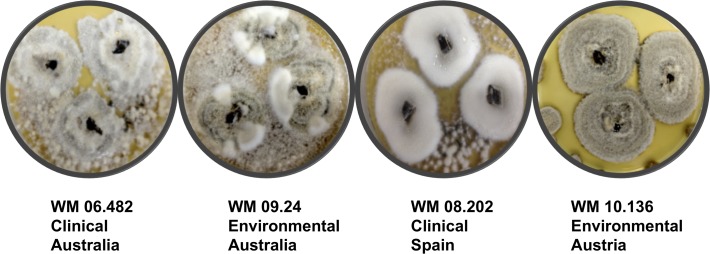
Colony morphology of the four different strains of *S*. *aurantiacum* growing on Sabouraud dextrose agar plates after 14 days of incubation.

The first phenotypic feature that separated the four *S*. *aurantiacum* strains was the appearance of colonies on Sabouraud’s agar. The color of the colonies varied from greyish white in WM 06.482, white in WM 08.202, suede-like in WM 10.136 and brownish-white in case of WM 09.24 (Fig. 1). All strains were slow-growing and produced a light yellow pigment on the reverse of the agar plates after 14 days of incubation.

**Fig 2:**
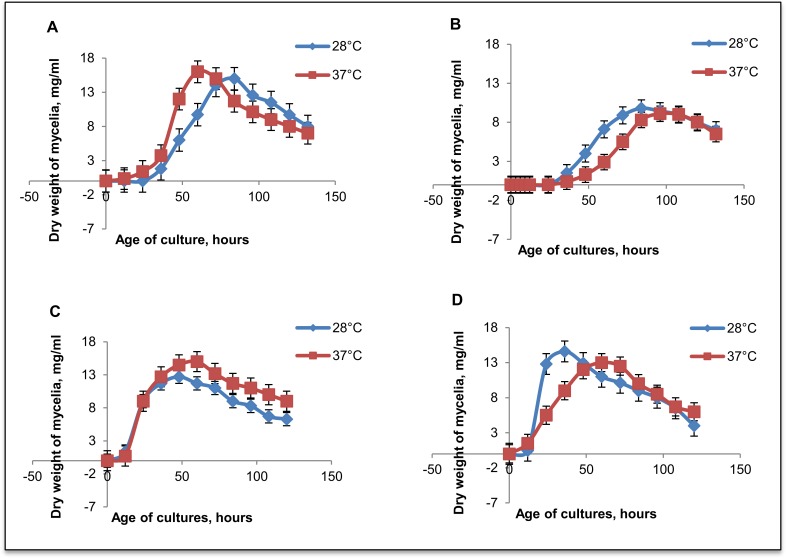
Growth of the *S*. *aurantiacum* strains in liquid culture in Sabouraud’s broth, measured as change in the mycelial dry weight (mg/ml) over time. **A) WM 06.482, B) WM 08.202, C) WM 09.24 and D) WM 10.136.** Each experiment was repeated in triplicate, with bars representing ±1.25 standard errors, and 95% confidence interval.

Submerged cultures were grown in triplicate to establish a growth pattern for each strain. Growth of *S*. *aurantiacum* can be divided into four phases: lag, 0 to 36 hr; log, 36 to 48 hr; stationary, 48 to 72–84 hr and death after 84 hr (Fig. 2). A shorter lag phase (0–24 hours) was observed for the environmental strains (WM 09.24 and WM 10.136) whereas the clinical strains (WM 06.482 and WM 08.202) showed a longer lag phase, with the first significant change in the dry weight after 48 hours of incubation. As a general trend, the average mycelial dry weight reached its maximum in the log phase and decreased thereafter. WM 08.202 (type strain) was the slowest growing *S*. *aurantiacum* strain and also produced least biomass as compared to the others (Fig. 2).

Temperature had an impact on the growth of *S*. *aurantiacum* strains. WM 06.482 (clinical isolate) and WM 09.24 (environmental isolate) exhibited better growth at 37°C (as seen in Fig. 2), but were able to adapt to the lower temperature (28°C). The slowest growing strain WM 08.202 (clinical isolate) showed equal mycelial dry weights at 28°C and 37°C and strain WM 10.136 (environmental isolate) preferred 28°C, at which temperature the growth was fast and efficient. All four strains tested were able to adapt to the mammalian body temperature *i*.*e*. 37°C. Adaptation to various culture conditions, especially tolerance of mammalian temperatures (37°C) is a well-established phenomenon in other opportunistic fungal pathogens such as *Cryptococcus neoformans* [30].

### Ranking the *S*. *aurantiacum* strains according to virulence

**Fig 3:**
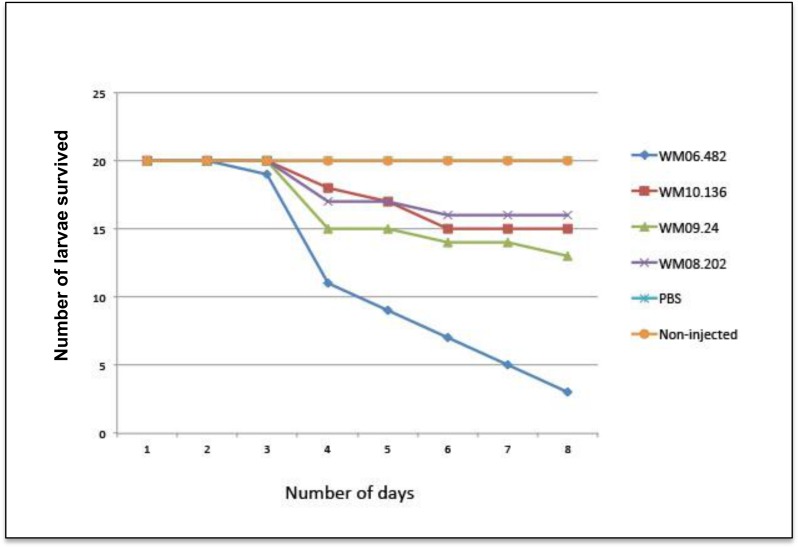
Survival of *G*. *mellonella* larvae infected with different strains of *S*. *aurantiacum*.

Virulence levels of the four *S*. *aurantiacum* isolates were explored using the *G*. *mellonella* larvae invertebrate model that has been previously used to assess the virulence of different strains of the human fungal pathogen *Candida albicans* [31]. Survival of larvae infected with the different strains is shown in Fig. 3. No larval death was observed in any of the control groups *i*.*e*. non-treated larvae and larvae inoculated with PBS.

As seen in Fig. 3, all *S*. *aurantiacum* strains were able to kill larvae; WM 06.482 (clinical isolate) was the most virulent among the strains tested, as the majority (85%) of the larvae failed to survive after eight days of infection and WM 08.202 (type strain isolated from a wound exudate) had the least effect on larval mortality (20%) The environmental strains, namely WM 09.24 and WM 10.136, killed approximately 40% and 25% of the larvae, respectively, within eight days.

While the *G*. *mellonella* model did clearly separate the strains with highest (WM 06.482) and lowest virulence (WM 08.202 and WM 10.136), the nature of WM 09.24 is less clear. Nevertheless, from this analysis, WM 09.24 was the second most virulent strain of the four *S*. *aurantiacum* strains tested. Similar to previous studies [11, 32] the virulence levels observed for different *S*. *aurantiacum* strains used in this study were independent of the origin of the strain as the environmental isolate WM 10.136 had similar virulence properties as the clinical strain WM 08.202.

### Carbon utilization by *S*. *aurantiacum* strains

The virulence levels of *S*. *aurantiacum* strains can be attributed to the physiological differences [33], analysis at the phenotypic level can help to further understand the mechanisms of pathogenicity in this organism [34, 35]. Thus, utilization of a variety of nutrients (especially carbon sources) by the four *S*. *aurantiacum* strains was tested using an automated high throughput Biolog assay. The opm package for R was used for data analysis as it provides a range of benefits such as visualization and curve parameter estimation, metadata management, customizable plots and automated generation of tabular reports [36].

**Fig 4:**
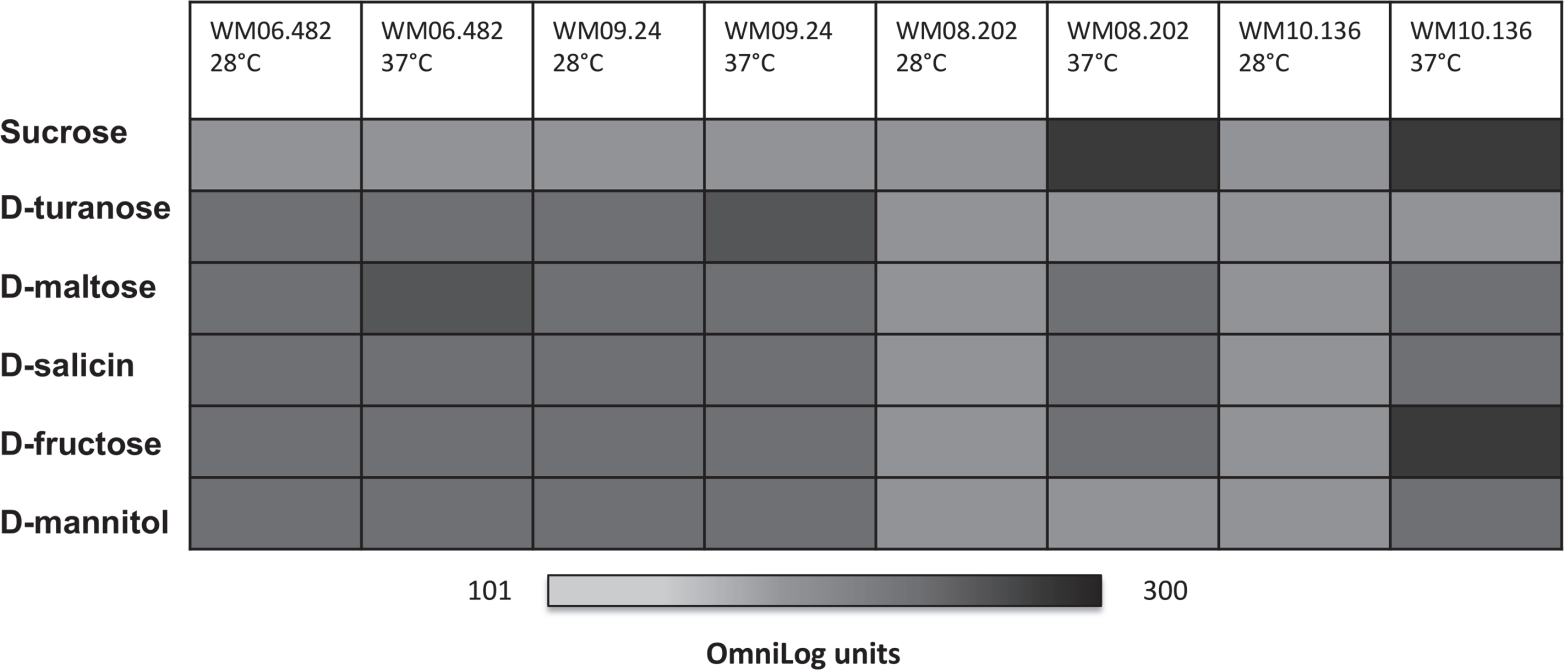
Different sugar utilization patterns of low and high virulent strains. OmniLog units with a minimum value of 100 depict a positive phenotype.

All four strains were able to respire on 54 out of the 94 substrates displayed on the GenIII microplates, including a range of sugars, a few amino acids and hexose acids at either 28 or 37°C. Temperature-based differences were observed specifically, when comparing the utilization of the most common substrate groups such as carbohydrates, carboxylic and amino acids. For example, maltose, D-salicin, D-fructose and lactose were utilized by two *S*. *aurantiacum* strains namely WM 08.202 and WM 10.136 only at 37°C. This is different from both WM 06.482 and WM 09.24, which could respire on these substrates at both 28 and 37°C thereby showing a similar metabolic response to the cultivation temperature (Fig. 4). Adaptation to the imposed temperature conditions has been described as an essential attribute of many highly virulent pathogens [37, 38].

Carbon acquisition and metabolism is central to the virulence and persistence of many lung pathogens and can be used to distinguish different strains within the species [34]. For example, difference in assimilation of five different carbon sources (ribitol, L-arabinitol, sucrose, maltose and ribose) was used to discriminate between the species. *S*. *aurantiacum* was distinguished from other members of the complex based on its inability to use sucrose as a substrate for growth [4]. In this study, two strains of *S*. *aurantiacum* (WM 06.482 and WM 09.24) were unable to metabolize sucrose but instead showed higher cellular respiration on its isomeric form turanose. While the outcome may seem surprising at the first glance, however there are previous studies proposing utilization of turanose as a potential indicator for virulence [39]. Turanose is known to promote high level of mycelial growth in the plant pathogen *Fusarium virguliforme*, and activates defense responses in higher plants suggesting a possible association between turanose assimilation and pathogenic properties [40–42]. High respiration rates on sucrose in WM 08.202 and WM 10.136 point towards the presence of a functional sucrose utilization pathway.

### Correlation of growth and respiration assays

**Fig 5:**
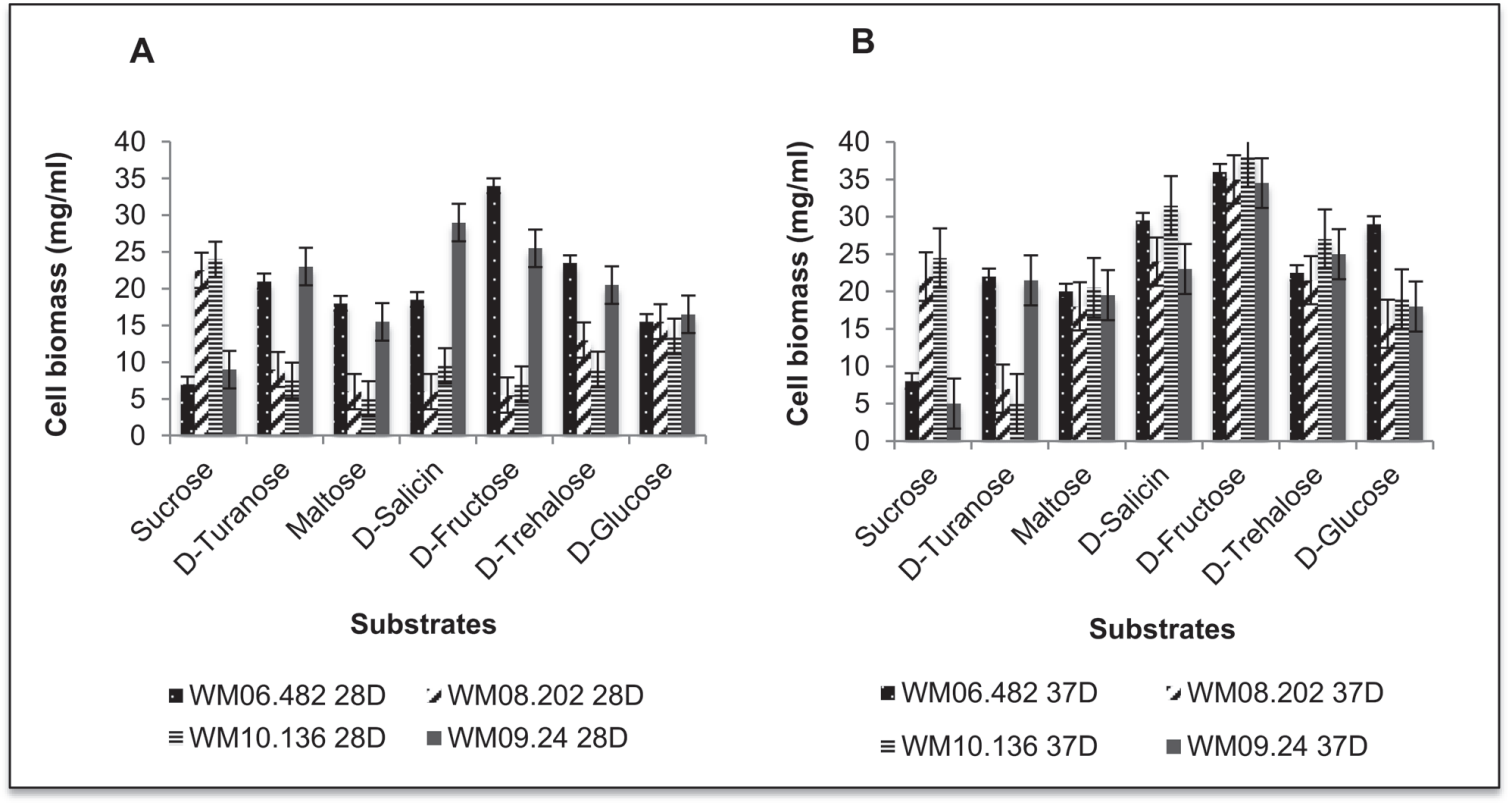
Relative growth of *S*. *aurantiacum* in minimal medium with different carbon sources, measured as average dry weight of mycelia at a) 28°C and b) 37°C. A different pattern is assigned to each strain as shown in the key below. The graphs clearly distinguish between the growth patterns of high and low virulent strains on selected carbon sources at both temperatures. (Weight of the inoculum was 10 mg/ml. Therefore biomass value above this limit was considered as significant growth). Each experiment was repeated in triplicate, with bars representing ±1.25 standard errors, and 95% confidence interval.

Cell respiration generally correlates with cell growth. In order to confirm the cellular respiration results obtained in the Biolog assay, growth of *S*. *aurantiacum* strains was determined in liquid cultures on a minimal medium supplemented with a panel of selected carbon compounds, incubated at 28°C (Fig. 5A) and 37°C (Fig. 5B). In accordance with the PM data, WM 06.482 and WM 09.24 showed growth on maltose, D-trehalose, D-salicin and D-fructose at both temperatures (28 and 37°C), whereas WM 08.202 and WM 10.136 were able to grow on these substrates only at high temperature (37°C).

**Fig 6:**
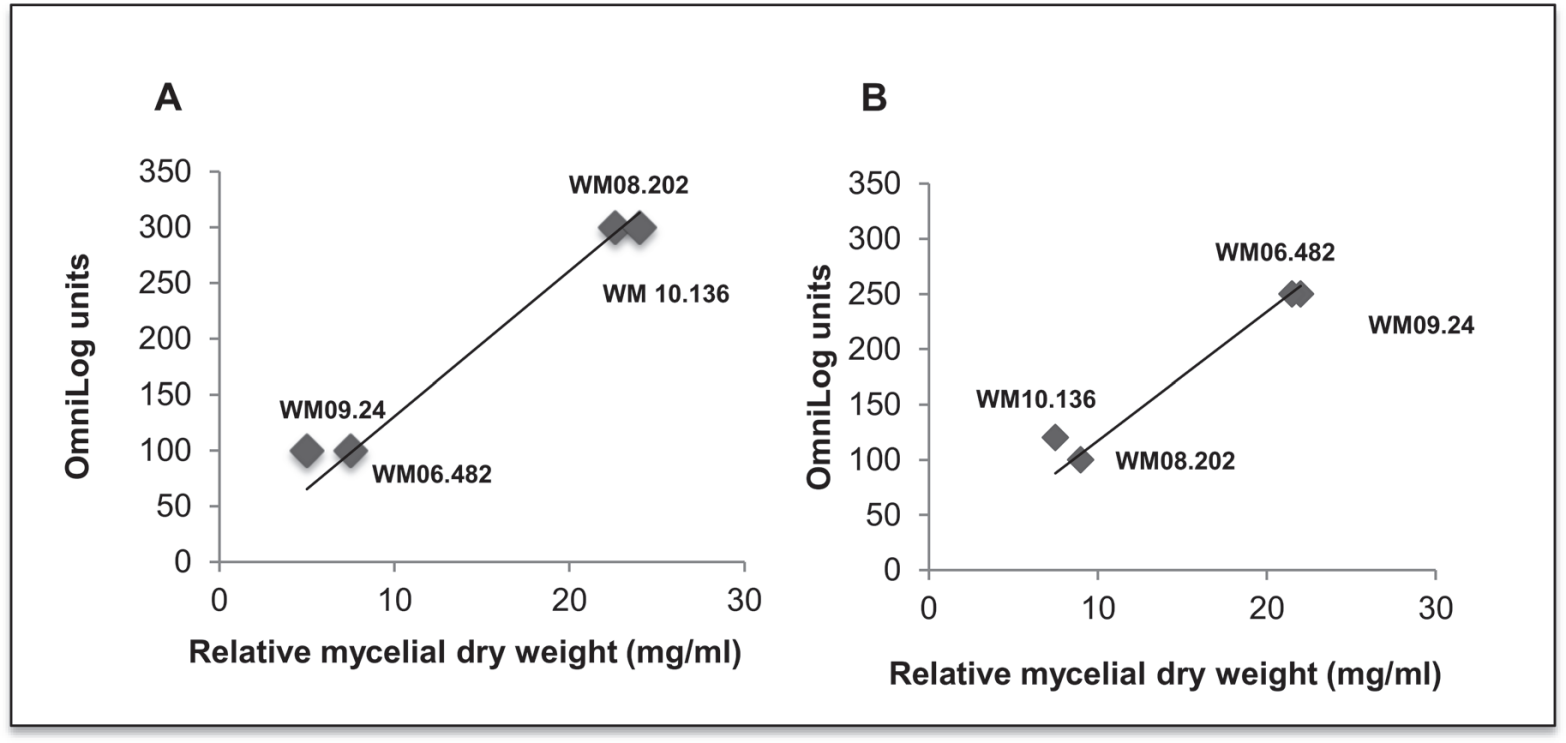
Correlation between cell growth and cell respiration for sucrose and turanose for *S*. *aurantiacum* at 37°C. Cell biomass was calculated for all the strains grown in sucrose and turanose supplemented minimal media and plotted against the respiration rates (OmniLog units). a) 1mM sucrose; b) 1mM turanose.

Growth in the shake flasks was consistent with cell respiration on all six tested substrates as shown by plotting the cell biomass against the OmniLog units. Examples of this correlation at 37°C are shown in Fig. 6A for sucrose and Fig. 6B for turanose. *S*. *aurantiacum* strains WM 06.482 and WM 09.24 that showed no respiration on sucrose in the Biolog assay also did not show any growth in the minimal medium supplemented with sucrose. Instead, these strains grew well on turanose which is in accordance with efficient respiration measured in the PM assay on this substrate at 37°C. In contrast, the established low virulence strains WM 08.202 and WM 10.136 exhibited good respiration and growth on sucrose. Overall, the growth assays in liquid media were concordant with the Biolog phenotype microarray data, thus validating the respiration assay and illustrating its usability as a high-throughput phenotype assay for *Scedosporium*. These findings suggest that common characteristics were shared between two groups of *S*. *aurantiacum* strains WM 06.482 and WM 09.24; and WM 08.202 and WM 10.136 as reflected in their substrate utilization patterns.

### Resistance to selected chemicals

PM analysis revealed that all four strains of *S*. *aurantiacum* were highly halotolerant as high respiration rates were observed on high sodium chloride concentrations (4% and 8%). The salt tolerance capacity of *S*. *aurantiacum* is close to the optimum salt concentration required for the growth of the halophilic black yeast *Hortaea werneckii*, the most salt tolerant eukaryotic organisms reported to date [43]. Saline resistance could be one of the reasons for the persistence of *S*. *aurantiacum* in the salt-rich airway fluid of CF patients [44]. CF patients have abnormal salt transport across the airway epithelium, which causes defective mucociliary clearance and reduced clearance of the infectious agents [45]. Similarly, resistance of *S*. *aurantiacum* to other chemical treatments such as nalidixic acid and low pH under different temperature conditions can explain their pervasive nature and ability to survive under extreme environmental conditions [46].

### The effect of amino acids on growth

The phenotype assays revealed some hexose acids, carboxylic acids, esters and fatty acids that resulted in slow or no growth. Examples of such compounds are aspartic acid, D-serine, L-histidine, L-pyroglutamic acid, D-galacturonic acid, L-galactonic acid-g-lactose, D-gluconic acid, D-glucuronic acid, mucic acid, D-saccharic acid, D-lactic acid methyl ester, a-keto-glutaric acid, D-malic acid and sodium formate. Some of these compounds such as D-lactic methyl ester that caused slow growth or no growth at all have been considered as inhibitors of the spore germination process e.g. in *Hypocrea jecorina* [21]. Given the high resistance of *S*. *aurantiacum* to many antifungal agents, similar combined strategies can be devised for screening the potential inhibitors against this pathogenic fungus.

### Metabolic reconstructions using the *S*. *aurantiacum* draft genomes

**Fig 7:**
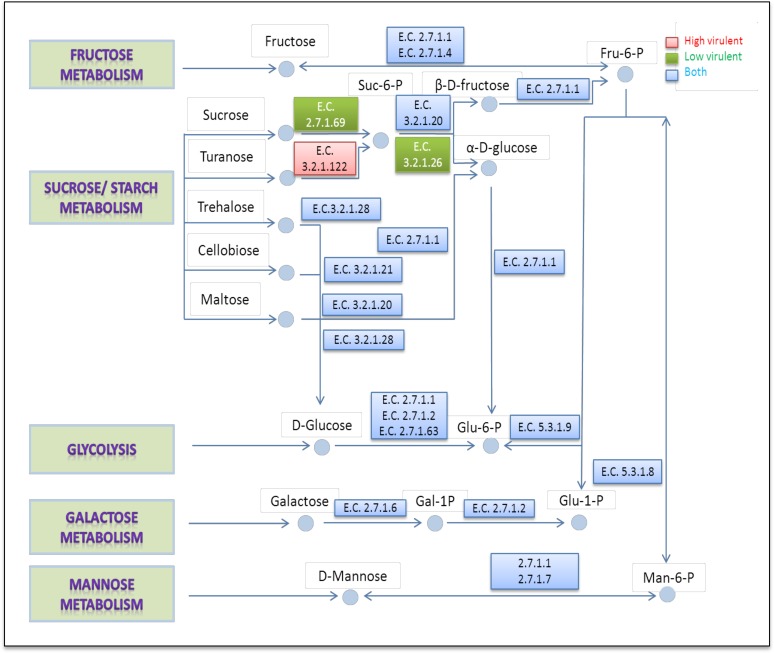
Schematic overview of various metabolic pathways present in *S*. *aurantiacum* obtained after superimposition of PM data with the assembled genome.

Metabolic reconstructions were carried out for all the four *S*. *aurantiacum* strains by ascribing the observed phenotypic differences to the presence or absence of certain enzyme encoding genes in a particular carbohydrate utilization pathway. The main metabolic pathways found in the analysis were then used for the generation of biochemical maps (Fig. 7) that show the network of genome-encoded enzymes catalyzing the metabolism of different carbohydrates in the four different *S*. *aurantiacum* strains studied in this work. Five major carbohydrate metabolism pathways were revealed in the genome-wide analysis of *S*. *aurantiacum*, namely sucrose, fructose, mannose and galactose metabolism and glycolysis (Fig. 7).

Sucrose metabolism can involve either one-step or a two-step reaction depending on the enzymes involved in the overall process. In a two-step reaction common in many filamentous fungi, sucrose is converted to sucrose-6-phosphate by the phosphotransferase enzyme and ultimately to the end product glucose in a reaction catalyzed by invertase [47, 48]. Alternatively, sucrose can be directly hydrolyzed to glucose solely by invertase. Based on the metabolic pathways reconstructed from genome data, only the low virulence strains WM 08.202 and WM 10.136 seemed to possess both the phosphotransferase (E.C. 2.7.1.69) and invertase (E.C. 3.2.1.26) enzymes which allowed them to utilize sucrose for growth and respiration.

On the other hand, inability of WM 06.482 and WM 09.24 to grow/respire on sucrose (as seen in Fig. 4 and 6) can be explained by the absence of sucrose-hydrolysing enzymes as revealed by the genome correlation. The assimilation of turanose in these strains can be attributed to the presence of phospho-alpha-glucosidase (E.C. 3.2.1.122) which was not found in the low virulence strains. The other three metabolic pathways studied including fructose, galactose and mannose metabolism were similar in all four *S*. *aurantiacum* strains. Thus phenotypic testing allowed us to specifically search for the genetic factors underpinning the phenotypes of different strains of *S*. *aurantiacum*.

In principle, the presence or absence of respective enzymes in different *S*. *aurantiacum* strains can be verified by amplification of the gene sequences from the genomic DNA. However, considering the non-availability of a fully annotated *S*. *aurantiacum* genome and the limited overall sequence homology (~40%) for the identified gene sequences between *S*. *aurantiacum* and the reference organisms, this methodology can lead to inconclusive results. Nevertheless, the ability of the strains to metabolize the discussed carbohydrates is a strong, yet indirect indication for the presence of genes encoding the required enzymes [49].

## Conclusions

We have used morphological, physiological and metabolic assessment to characterize four different *S*. *aurantiacum* strains exhibiting different virulence levels. The analysis helped to identify metabolic differences between two groups of *S*. *aurantiacum* strains, WM 06.482 and WM 09.24; and WM 08.202 and WM 10.136. Correlation of the genome information with the metabolic assessment assisted in exposing five putative carbohydrate metabolism pathways of which sucrose and D-turanose utilization different between the above *S*. *aurantiacum* groups. While classification of the environmental strain WM 09.24 as a high or low virulence strain was not straightforward from the *Galleria mellonella* assay, it behaved similar to the high virulent strain WM 06.482 for which the virulence has also been established in a mouse model. Therefore we group WM 09.24 together with WM 06.482, which leads us to speculate on metabolic differences between high and low virulence strains, such as ability to utilize D-turanose. The differences can be investigated further with fully annotated genomes available in the near future.

## Supporting Information

S1 Fig Layout of biolog GenIII plate. Layout of the Biolog GenIII plate depicting various conditions/substrates used to detect substrate utilization of *S*. *aurantiacum* strains WM06.482, WM08.202, WM10.136 and WM09.24. The various substrates listed can be categorized as follows: 1) Control: A1. 2) Sugars: A2-A9, B1-B9 and C1-C9. 3) Hexose phosphates: from D06 and D07. 4) Amino acids: from E1-E9. 5) Hexose acids: from F1-F9. 6) Carboxylic acids, esters and fatty acids: G1-G9 and H1-H9. 7) Acidic pH: A11 and A12. 8) NaCl: B10-B12. 9) Lactic acids: C10. 10) Reducing agents: F11 and F12. 11) Gram negative/gram positive: F10 and G10.
(TIFF)
