# Supplementary figures and images for: Phenotypic Profiling of Scedosporium aurantiacum, an Opportunistic Pathogen Colonizing Human Lungs
Source: PLoS One. 2015 Mar 26;10(3):e0122354. doi: 10.1371/journal.pone.0122354 (PMC4374879; doi:10.1371/journal.pone.0122354)

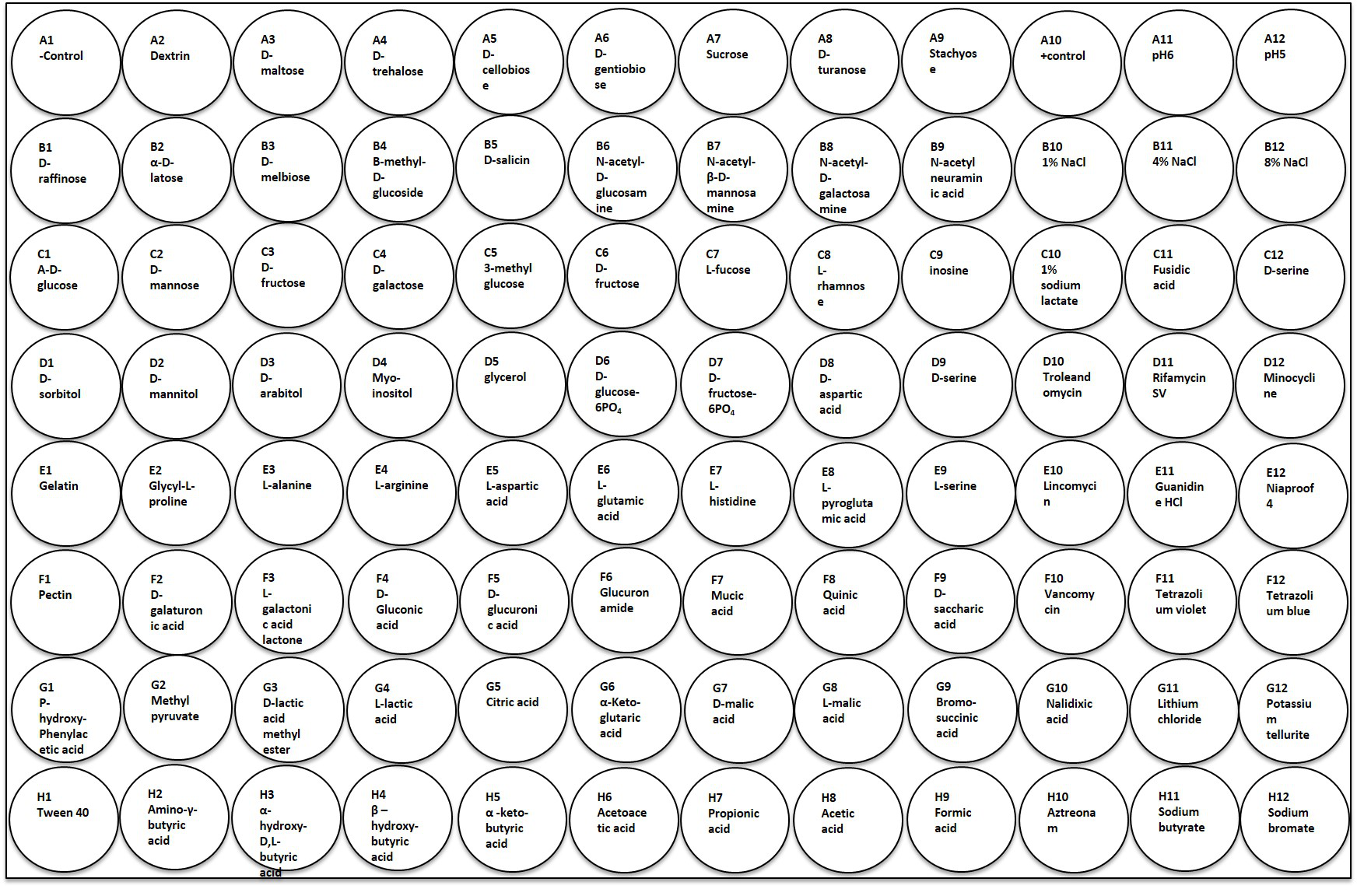

Supplement: S1 Fig — Layout of the Biolog GenIII plate depicting various conditions/substrates used to detect substrate utilization of S. aurantiacum strains WM06.482, WM08.202, WM10.136 and WM09.24. The various substrates listed can be categorized as follows: 1) Control: A1. 2) Sugars: A2-A9, B1-B9 and C1-C9. 3) Hexose phosphates: from D06 and D07. 4) Amino acids: from E1-E9. 5) Hexose acids: from F1-F9. 6) Carboxylic acids, esters and fatty acids: G1-G9 and H1-H9. 7) Acidic pH: A11 and A12. 8) NaCl: B10-B12. 9) Lactic acids: C10. 10) Reducing agents: F11 and F12. 11) Gram negative/gram positive: F10 and G10. (TIFF) [file pone.0122354.s001.tiff]
